# Determinants of Non-Receipt of Guideline-Concordant Treatment in Pancreatic Cancer: A Classification Tree Analysis

**DOI:** 10.1245/s10434-026-20369-x

**Published:** 2026-08-12

**Authors:** Manish Tripathi, Ioannis Liapis, Nritya Nair, Marcus Sirianno, Alexis Kennedy, Smita Bhatia, Annabelle L. Fonseca

**Affiliations:** 1 Institute of Cancer Outcomes and Survivorship, University of Alabama at Birmingham, Birmingham, AL USA; 2 Collat School of Business, The University of Alabama at Birmingham, Birmingham, AL USA; 3 Department of Surgery, University of Alabama at Birmingham, Birmingham, AL USA; 4 Edward Via College of Osteopathic Medicine, Auburn, AL USA

## Abstract

**Background:**

Non-receipt of guideline-concordant treatment (GCT) in pancreatic ductal adenocarcinoma (PDAC) is associated with worse outcomes. Studies have identified associated factors, but less is known about how these factors co-occur within patient subgroups at the time of presentation to oncology care.

**Methods:**

We conducted a retrospective cohort study of patients with PDAC treated at two academic centers. Candidate variables available at presentation were selected *a priori* across demographic, clinical, socioeconomic, and healthcare utilization domains. Classification and regression tree (CART) models were used to identify conditionally defined subgroups associated with non-receipt of GCT. Models were developed using an 80/20 training/test split with repeated cross-validation.

**Results:**

Of 1040 patients, 337 (32.4%) did not receive GCT. In both unpruned and pruned models, Area Deprivation Index (ADI) defined the initial split. Among patients with ADI ≥7, non-receipt of GCT was more common among those entering care through the emergency department, with further stratification by race, social support, and timeliness of care. Among patients with ADI <7, stratification was based on age, social support, and performance status. In sensitivity analyses excluding age, ADI remained the initial split, with downstream stratification based on cancer screening, insurance, social support, and albumin. The model demonstrated an area under the receiver operating characteristic curve (AUROC) of 0.849 on the training set and 0.662 on the independent test set.

**Conclusion:**

Non-receipt of GCT in PDAC is associated with distinct clinically identifiable patterns involving socioeconomic context, access to care, and patient vulnerability at presentation, highlighting potential targets for early intervention.

**Supplementary Information:**

The online version contains supplementary material available at https://doi.org/10.1245/s10434-026-20369-x.

Pancreatic ductal adenocarcinoma (PDAC) is projected to become the second leading cause of cancer-related mortality in this decade.^1^ Despite advances in systemic therapy and surgical technique, overall survival remains poor, with a current 5-year overall survival of 13%.^2^ The National Comprehensive Cancer Network (NCCN) Clinical Practice Guidelines provide evidence-based, stage-specific treatment recommendations,^3^ and treatment that is adherent to these guidelines (i.e., guideline-concordant treatment [GCT]) is associated with improved survival across all disease stages.^4,5^

However, between 30% and 50% of patients with PDAC do not receive GCT.^6–8^ Studies have identified several factors associated with non-receipt of GCT, including age, race, comorbidity burden, and socioeconomic disadvantage.^9–12^ While these factors are well described, they are typically evaluated as independent predictors using traditional models, which do not reflect how barriers to care co-occur and vary across patient subgroups in clinical practice. In real-world cancer care delivery, structural barriers may influence or amplify frailty or clinical vulnerability both before and during treatment delivery. Understanding how these factors cluster and interact is important to identify patients at risk and to inform targeted interventions. Classification and regression tree (CART) methods provide an approach to identify conditionally defined subgroups based on combinations of variables, allowing for the exploration of hierarchical relationships in a clinically interpretable format.

In this dual-institution retrospective study of patients with PDAC treated at two academic safety-net facilities in the southeastern United States, we applied a CART approach to examine patterns of stratification associated with non-receipt of GCT. Our objective was to identify clinically interpretable subgroups defined by combinations of demographic, clinical, socioeconomic, and access-related factors available at the time of presentation for oncology care.

## Methods

### Study Design and Patient Population

We conducted a retrospective cohort study of adult patients with PDAC treated between 2018 and 2022 at two academic tertiary facilities in the Southeastern United States. Both institutions serve as regional referral centers and safety-net hospitals serving a large, socioeconomically diverse population.

Patients were identified using *International Classification of Diseases, Tenth Revision* (ICD-10) codes C25.0–C25.3 and C25.7–C25.9. Patients with missing data on key demographic, staging, or treatment variables were excluded from the analysis. For patients who received portions of care at both institutions, data were reconciled to avoid duplication. The study was approved by the institutional review boards at both institutions.

### Outcome Definition

The primary outcome was receipt of GCT, analyzed as a binary variable, with non-receipt as the outcome of interest. GCT was defined based on NCCN guidelines as receipt of stage-appropriate cancer-directed therapy^3^ and adjudicated using detailed manual chart review incorporating disease stage, anatomic resectability, multidisciplinary treatment recommendations, documented contraindications, disease progression, and patient goals of care when documented. For patients with stage I or II PDAC, GCT was defined as receipt of systemic chemotherapy (in neoadjuvant or adjuvant sequence) and curative-intent surgical resection unless there was documentation of contraindication due to comorbidities that precluded surgical resection or progression of disease on chemotherapy. For patients with stage III disease, GCT was defined as receipt of systemic chemotherapy with or without subsequent radiation or surgery. For patients with stage IV disease, GCT was defined as receipt of systemic chemotherapy, unless there was documentation of clinical status that precluded receipt or a palliative care consult and patient decision to forego treatment.

Disease stage was determined using the best available clinical information available at the time treatment decisions were made, supplemented by subsequent pathological findings as necessary. American Joint Committee on Cancer (AJCC) staging criteria applicable during the study period were used. Anatomic resectability (resectable, borderline resectable, locally advanced) was collected separately and incorporated into outcome adjudication.

Receipt of GCT was confirmed through comprehensive manual chart review of each patient’s longitudinal treatment course. Review included medical, surgical, and radiation oncology documentation; all procedural, imaging, and pathology reports; nursing, social work, navigation, and other ancillary provider documentation; and outside records. Patients were not classified as having non-GCT because of isolated treatment delays, treatment modifications, or discontinuation after disease progression when management remained consistent with guideline-directed care. For patients who did not receive GCT, the primary proximate reason for non-receipt was assigned after review of the complete longitudinal record. When multiple contributing factors were present, patients were classified according to the dominant factor judged to have most directly prevented initiation or completion of stage-appropriate therapy.

### Variables and Data Preparation

Candidate variables were selected *a priori* based on clinical relevance, prior literature, and availability at the time of presentation to oncology care. Variables were grouped into the following four domains.

1. Demographics: age at diagnosis, sex, race/ethnicity, and marital status.
2. Clinical factors: Eastern Cooperative Oncology Group (ECOG) performance status, Charlson Comorbidity Index, body mass index (BMI), albumin level, smoking status, and alcohol and substance use.
3. Socioeconomic and access-to-care metrics: insurance status, type of social support, driving distance to treatment center, rural versus urban county of residence, presence and type of primary care provider (PCP), up-to-date cancer screening, and the State Area Deprivation Index (ADI).
4. Healthcare utilization: location of diagnostic workup initiation (i.e., emergency department [ED] vs. outpatient primary care or other physician), time from symptom onset to initiation of diagnostic workup, and time from diagnosis to initial oncology appointment.

State ADI, a decile ranking, was used as a measure of neighborhood-level disadvantage. ADI is a validated composite index derived from the U.S. Census data incorporating factors under the domains of income, education, employment, and housing quality, with higher values indicating greater deprivation.^13^ For descriptive analyses, ADI was categorized into low (1–3), intermediate (4–6), and high (7–10) deprivation. For CART modeling, ADI was included as a continuous variable, allowing data-driven determination of optimal split points.

To reflect a clinically relevant decision point, analyses were restricted to variables available at or before the time of initial presentation to oncology care, allowing evaluation of factors identifiable early in the treatment pathway. Accordingly, variables not consistently available at the initial oncology consultation (e.g., disease stage) were not considered candidate variables for CART model development. Although disease stage and anatomic resectability were incorporated into adjudication of the primary outcome, they were intentionally excluded from the CART analysis, which was designed to identify patients at risk for non-receipt of GCT at initial presentation.

To enhance clinical interpretability and prevent model overfitting, the following continuous variables were categorized into clinically meaningful intervals: Age was grouped by decade, driving distance by 50-mile increments, and time intervals in weeks. Albumin and BMI were categorized using standard clinical thresholds. Missing data were minimal (<5% across variables), and no imputation was performed. All candidate variables were included in model development.

### Statistical Analysis

Descriptive statistics were used to summarize baseline characteristics. Categorical variables were compared using chi-squared tests, and continuous variables were compared using non-parametric tests as appropriate.

A CART approach was used to examine hierarchical relationships between candidate variables in relation to non-receipt of GCT. This method recursively partitions the dataset into subgroups based on predictor variables, generating a tree structure that facilitates identification of conditionally defined subgroups in a clinically interpretable format. The dataset was randomly partitioned into a training set (80%) and an independent test set (20%) using stratified sampling to preserve the distribution of the primary outcome.

An initial unpruned tree was constructed using the training set to explore higher-order interactions among variables. Tree construction was performed using predefined constraints to promote interpretability and reduce overfitting to allow for a maximum tree depth of 12, a minimum of 15 samples required to perform a split, and a minimum of 10 samples per terminal node. Splits were selected based on reduction in Gini impurity. For continuous variables, split points were determined algorithmically.

To reduce overfitting and improve generalizability, cost-complexity pruning was performed using repeated 5-fold cross-validation. The optimal pruned tree was selected based on cross-validated performance. Variable importance was assessed using the total reduction in Gini impurity, and the most influential features were retained for final modeling. The final model was evaluated on the independent test set. Model discrimination was assessed using the area under the receiver operating characteristic curve (AUROC). Sensitivity, specificity, and accuracy were calculated at the Youden-optimal threshold. A sensitivity analysis excluding age was performed to evaluate the stability of the tree structure and assess whether observed patterns were dependent on age as a dominant stratifying variable.

### Statistical Software

All analyses were conducted using Python (version 3.12; Python Software Foundation) using standard statistical and machine learning libraries, including pandas and scikit-learn.

## Results

### Patient Characteristics

A total of 1040 patients with PDAC were included, of whom 337 (32.4%) did not receive GCT. Overall, 30% presented with stage I, 20% with stage II, 23% with stage III, and 27% with stage IV disease. Baseline characteristics stratified by receipt of GCT are shown in Table 1. Patients who did not receive GCT were older, with rates of non-GCT increasing from 23% in patients aged <50 years to 56.1% in those aged ≥80 years (p <0.001). Non-receipt of GCT was also more common among female (36% vs. 29%, p=0.02), Black (43.6% vs. 29%, p <0.001), and unmarried (40.3% vs. 26.8%, p<0.001) patients. Receipt of GCT also differed by disease stage, with non-receipt occurring in 38%, 36%, 26%, and 29% of patients with stage I, II, III, and IV disease, respectively (p= 0.004).

**Table 1:** Baseline characteristics stratified by receipt of guideline-concordant treatment (GCT)

Characteristic	GCT n=703 (67.6)	No GCT n=337 (32.4)	*p*-value
**Demographic variables**			
*Age, years*			
<50	45 (77.6)	13 (22.4)	< 0.0001
50–59	131 (72.8)	49 (27.2)	
60–69	283 (70.6)	118 (29.4)	
70–79	201 (66.3)	102 (33.7)	
≥80	43 (43.9)	55 (56.1)	
*Sex*			
Male	377 (71.0)	154 (29.0)	0.02
Female	326 (64.0)	183 (36.0)	
*Race*			
White	517 (71.0)	211 (29.0)	0.0002
Black	146 (56.4)	113 (43.6)	
Other	13 (68.4)	6 (31.6)	
Declined/unknown	27 (79.4)	7 (20.6)	
*Marital status*			
Married	445 (73.2)	163 (26.8)	< 0.0001
Unmarried	258 (59.7)	174 (40.3)	
**Clinical variables**			
*ECOG score*			
0	177 (74.1)	62 (25.9)	< 0.0001
1	406 (70.1)	173 (29.9)	
≥2	86 (50.6)	84 (49.4)	
*Charlson Comorbidity Index*			
0	317 (73.2)	116 (26.8)	< 0.0001
1	255 (68)	120 (32)	
2	77 (56.2)	60 (43.8)	
≥3	54 (56.8)	41 (43.2)	
*AJCC clinical stage at treatment decision*			
I	190 (61.7)	118 (38.3)	0.004
II	132 (63.5)	76 (36.5)	
III	176 (74.3)	61 (25.7)	
IV	205 (71.4)	82 (28.6)	
*BMI category*			
Underweight	22 (55.0)	18 (45.0)	0.017
Normal	283 (70.2)	120 (29.8)	
Overweight	210 (69.1)	94 (30.9)	
Obese	148 (67.0)	73 (33.0)	
Morbidly obese	18 (52.9)	16 (47.1)	
*Albumin level*			
Normal	497 (73.1)	183 (26.9)	< 0.0001
Low	206 (57.2)	154 (42.8)	
*Smoking status*			
Current	130 (65.3)	69 (34.7)	0.03
Past	208 (70.7)	86 (29.3)	
Never	359 (67.6)	172 (32.4)	
*Alcohol consumption*			
Current – daily	45 (67.2)	22 (32.8)	0.24
Current – intermittently	227 (70.9)	93 (29.1)	
Past – daily	23 (62.2)	14 (37.8)	
*Substance abuse*			
Yes	38 (63.3)	22 (36.7)	0.48
No	472 (68.8)	214 (31.2)	
**Socioeconomic and access-to-care variables**			
*Insurance status*			
Private	276 (75.8)	88 (24.2)	0.001
Medicare	346 (62.2)	210 (37.8)	
Medicaid	24 (55.8)	19 (44.2)	
VA	23 (82.1)	5 (17.9)	
Uninsured	25 (73.5)	9 (26.5)	
*Social support*			
Spouse	317 (77.5)	92 (22.5)	< 0.0001
Family and friends	271 (62.6)	162 (37.4)	
None/unknown	115 (58.1)	83(41.9)	
*Primary care provider*			
Physician	516 (72.3)	198 (27.7)	< 0.0001
Advanced practice provider	87 (62.6)	52 (37.4)	
None	91 (51.7)	85 (48.3)	
*Up-to-date cancer screening*			
Yes	283 (76.5)	87 (23.5)	< 0.0001
No	420 (62.7)	250 (37.3)	
*Driving distance, miles*			
0–50	279 (67.4)	135 (32.6)	0.71
51–100	193 (67.5)	93 (32.5)	
101–200	170 (66.4)	86 (33.6)	
201–300	52 (73.2)	19 (26.8)	
≥301	9 (69.2)	4 (30.8)	
*Rural county*			
Yes	196 (66.9)	97 (33.1)	0.77
No	507 (67.9)	240 (32.1)	
*State ADI*			
Low	332 (77.4)	97 (22.6)	< 0.0001
Intermediate	219 (73.5)	79 (26.5)	
High	143 (47.8)	156 (52.2)	
**Healthcare utilization variables**			
*Location of cancer work-up initiation*			
Emergency department	274 (58.7)	193 (41.3)	< 0.0001
Outpatient by primary physician	410 (75.1)	136 (24.9)	
Weeks from diagnosis to oncology appointment	1.0 (0.0–3.0)	1.0 (0.0–4.0)	0.13
Weeks from symptoms to start of workup	3.0 (1.0–7.0)	4.0 (2.0–10.0)	< 0.0001

Data are presented as n (%) or median (interquartile range) unless otherwise indicated.

ADI, Area Deprivation Index; AJCC, American Joint Committee on Cancer; BMI, body mass index; ECOG, Eastern Cooperative Oncology Group; VA, Veterans’ Affairs.

Markers of socioeconomic disadvantage were associated with non-receipt of GCT. Patients residing in high deprivation areas (ADI ≥7) had higher rates of GCT non-receipt than those in intermediate- or low-deprivation areas (52.2% vs. 26.5% and 22.6% respectively, p <0.001). Similarly, patients covered with Medicaid or Medicare were less likely to receive GCT than those with private insurance (44.2% and 37.8% vs. 24.2%, p <0.001).

Patients who did not receive GCT were more likely to have an ECOG score ≥2 (49.4% vs. 28.7%, p <0.0001), a CCI score ≥2 (43.5% vs. 29.2%, p <0.0001) , and low albumin levels (42.8% vs. 26.9%, p <0.0001).

Non-receipt of GCT was more common among patients without a PCP or with a non-physician PCP (48.3% and 37.4% vs. 27.7%, p< 0.0001) and among patients not up-to-date on cancer screening (37.3% vs. 23.5%, p< 0.0001). Patients with a longer time from symptom onset to initial diagnostic workup were also more likely to have non-receipt of GCT (median 4.0 vs. 3.0 weeks, p<0.0001).

The most common reasons for non-receipt of GCT included clinical deterioration preventing completion of planned multimodal therapy (147/337, 43.6%), health system and access barriers (78/337, 23.1%), loss to follow-up during the treatment pathway (58/337, 17.2%), failure to initiate recommended therapy (46/337, 13.6%), and patient preference (8/337, 2.4%). Detailed reasons for non-receipt of GCT are shown in Supplementary Table 1. Reasons represent the principal proximate cause of non-receipt of GCT assigned after comprehensive longitudinal chart review. Because multiple contributing factors were frequently present, reasons were classified according to the single dominant factor judged to have most directly prevented initiation or completion of stage-appropriate therapy.

### Cohort Partitioning

The cohort was randomly divided into a training set (n=832, 80%) and an independent test set (n=208, 20%) using stratified sampling by outcome. The proportion of patients not receiving GCT was similar between the training and test sets.

### Unpruned Classification Tree

**Fig. 1.**
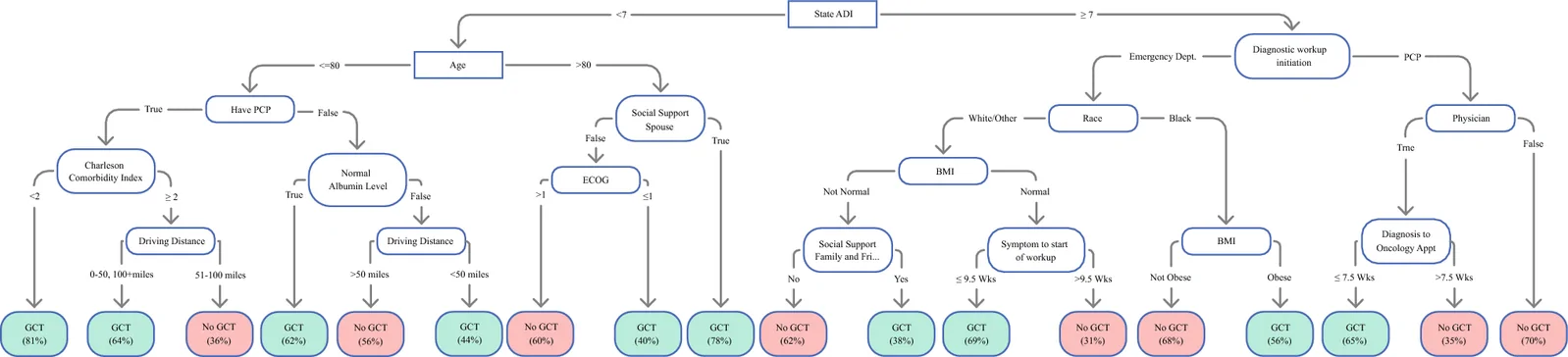
Hierarchical stratification of non-receipt of guideline-concordant treatment (GCT) in pancreatic ductal adenocarcinoma: unpruned classification tree. ADI, Area Deprivation Index; appt, appointment; BMI, body mass index; ECOG, Eastern Cooperative Oncology Group; PCP, primary care provider; wks, weeks; dept, department

The unpruned classification tree demonstrated ADI as the initial split at a threshold of 7 (Fig. 1). Among patients with ADI <7, subsequent stratification was based on age. In patients aged ≤80 years, the next split was based on the presence of a PCP, with higher rates of GCT among those with an established PCP. Within this group, comorbidity burden and driving distance further differentiated outcomes, with 81% of patients with a CCI <2 receiving GCT, compared with lower rates among those with higher comorbidity and greater travel distance. Among patients without a PCP, albumin level and distance to care were discriminators, with lower albumin and longer travel distance associated with reduced GCT receipt (~44–56%). Among patients aged >80 years, social support and performance status defined subsequent splits. Patients aged >80 years with spousal support had higher rates of GCT (78%), whereas those without spousal support were further stratified by ECOG performance status, with non-GCT rates of 60% with ECOG >1.

Among patients with ADI ≥7, the next split was based on location of diagnostic workup initiation. Patients entering care through the ED had lower rates of GCT than those entering through outpatient pathways. Within the ED group, race, BMI, and social support further stratified outcomes, with non-GCT rates of 31–68%. Among patients with diagnostic workup initiated by a PCP or other physician outside the ED, PCP type and time to oncology evaluation further differentiated outcomes, with non-GCT rates of 70% among those managed by a non-physician PCP. Patients with a physician PCP and a time interval from diagnosis to oncology appointment of <8 weeks had a GCT rate of 65%.

### Pruned Classification Tree

**Fig. 2.**
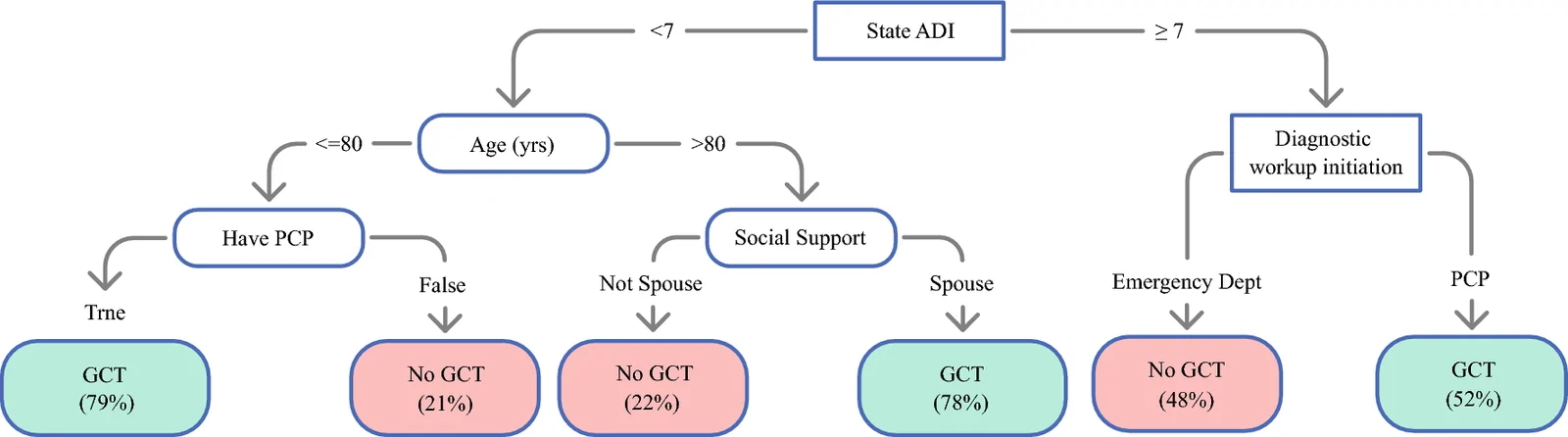
Pruned classification tree demonstrating patterns of stratification associated with non-receipt of guideline-concordant treatment (GCT). ADI, Area Deprivation Index; dept, department; PCP, primary care provider

The pruned classification tree retained ADI as the primary initial split at a threshold of 7 (Fig. 2). Among patients with ADI ≥7, the next split was based on location of diagnostic workup initiation, with patients entering care through the ED having a non-GCT rate of 48%. Among patients with ADI <7, the next split was based on age. Patients aged ≥80 years had lower rates of GCT, particularly among those without spousal support. Among patients aged <80 years, presence of a PCP further stratified outcomes, with GCT rates of 79% among those with a PCP. The final pruned tree resulted in three terminal nodes, with observed GCT rates ranging from 52% to 79% across subgroups.

### Sensitivity Analysis

**Fig. 3.**
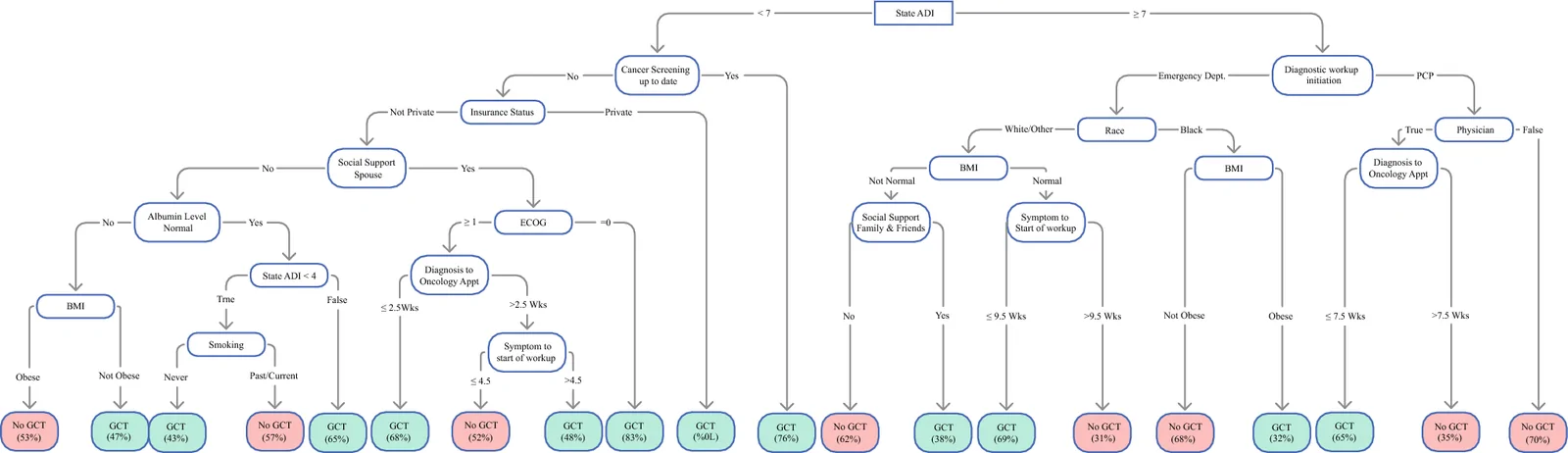
Sensitivity analysis excluding age: classification tree of factors associated with non-receipt of guideline-concordant treatment (GCT). ADI, Area Deprivation Index; BMI, body mass index; ECOG, Eastern Cooperative Oncology Group; wks, weeks; appt, appointment

In the sensitivity analysis excluding age (Fig. 3), the initial split remained ADI at a threshold of 7. Among patients with ADI <7, the next split was based on cancer screening status. Patients who were up to date on cancer screening had higher rates of GCT (76%), whereas those who were not were further stratified by insurance status, spousal support, and albumin levels. Patients with private insurance had higher GCT rates (70%), whereas non-GCT ranged from 43% to 57% among patients without private insurance and spousal support, with further stratification based on BMI, ECOG performance status, albumin level, and smoking status.

Among patients with ADI ≥7, the location of diagnostic workup initiation was the next split. Patients entering care through the ED were less likely to receive GCT, with non-GCT ranging from 62% to 68% based on race, BMI, and social support. Among patients with non-ED initiation of diagnostic workup, provider type and time to oncology evaluation further differentiated outcomes, with higher GCT rates among those with a physician PCP and a time interval from diagnosis to oncology appointment ≤7.5 weeks (65%). Patients with a non-physician PCP had a 70% rate of non-GCT.

### Model Performance and Risk Stratification

**Fig. 4.**
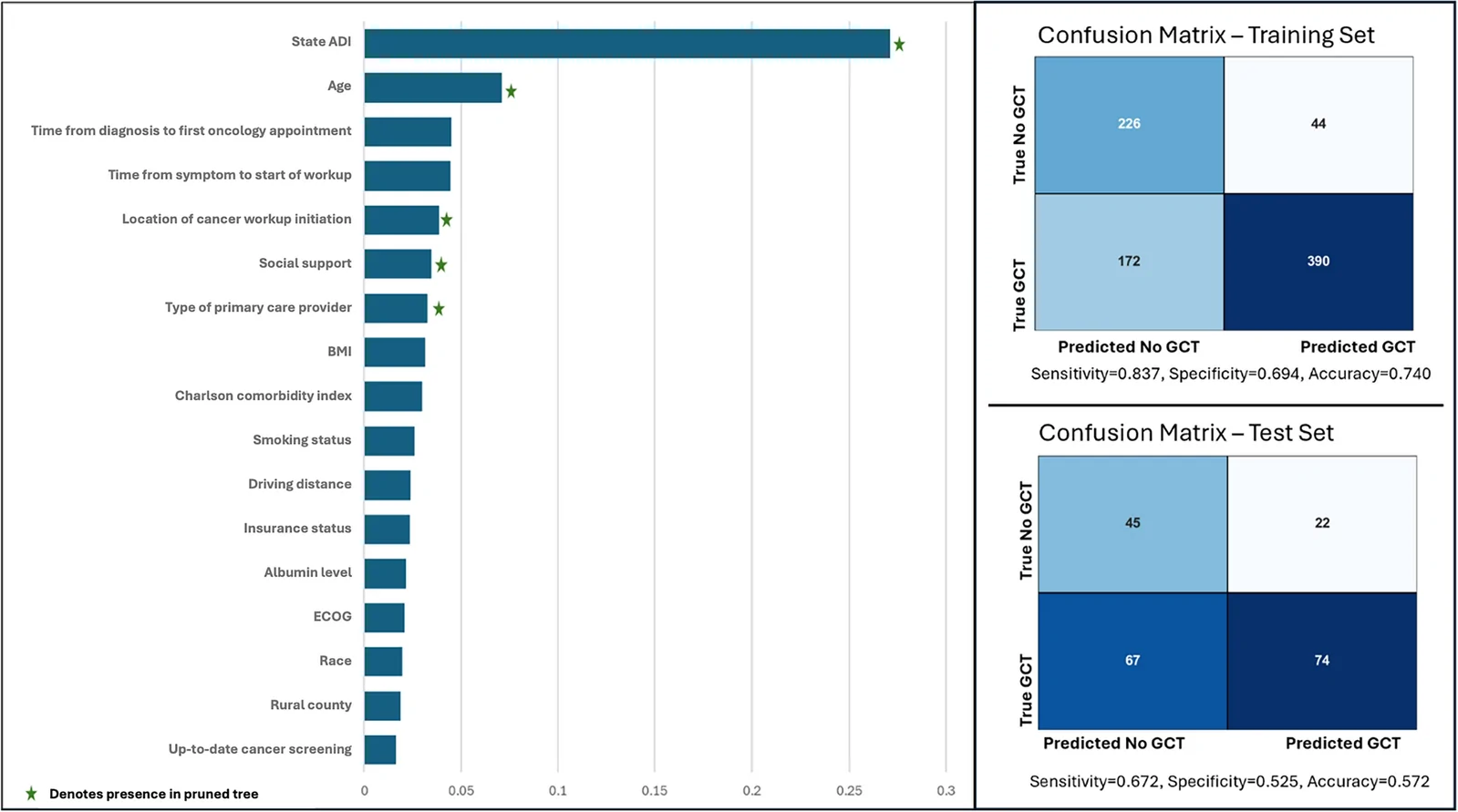
Variable importance and model classification performance. ADI, Area Deprivation Index; BMI, body mass index; ECOG, Eastern Cooperative Oncology Group; GCT, guideline-concordant treatment

Performance metrics for the CART models are presented for both the training and the independent test sets. On the training set, the model achieved an AUROC of 0.849, with a sensitivity of 83.7%, specificity of 69.4%, and accuracy of 74.0% at the Youden-optimal threshold of 0.333. When evaluated on the independent test set, model performance decreased, with an AUROC of 0.662. At the corresponding Youden-optimal threshold of 0.211, the model achieved a sensitivity of 67.2%, specificity of 52.5%, and accuracy of 57.2%. Variable importance and model performance are summarized in Fig. 4.

## Discussion

In this multi-institutional cohort of patients with PDAC, we identified consistent patterns of stratification associated with non-receipt of GCT across demographic, clinical, socioeconomic, and access-related factors. Neighborhood level deprivation, measured by ADI, was the primary stratifying variable across model specifications, with subsequent splits differing by deprivation level. Among patients residing in higher deprivation areas, non-receipt of GCT was more frequently observed in association with factors related to entry into the healthcare system, including ED-initiated workup. In contrast, among patients in low- and intermediate-deprivation areas, non-receipt was more commonly observed in association with age, functional status, and social support.

These findings extend prior work demonstrating disparities in pancreatic cancer care by illustrating how commonly identified factors may be conditionally related within specific patient subgroups. Prior studies have identified associations between GCT and age, comorbidity, race, and insurance status, typically using regression-based approaches.^6,11,14–17^ While these approaches quantify independent associations, they may not fully capture complex and hierarchical patterns that show how these factors co-occur in practice. Unlike conventional regression approaches that estimate average independent associations, CART identifies clinically recognizable combinations of variables that define patient phenotypes and sequential care pathways that are not readily visualized using traditional regression models, even when interaction terms are included. In this analysis, classification tree methods were used to describe conditionally defined subgroups, allowing visualization of how combinations of factors are associated with variation in receipt of GCT.

The consistent identification of ADI as the initial split across all models highlights the importance of neighborhood-level socioeconomic context. Among patients residing in higher deprivation areas, the location of initial diagnostic workup emerged as an early branching factor, with higher rates of non-receipt of GCT observed among those entering the healthcare system through the ED. This pattern was observed consistently across model specifications, including the sensitivity analysis excluding age. These findings suggest that, for patients in higher deprivation settings, early system-level barriers, such as fragmented care, delayed referral, or lack of established outpatient access, may play a dominant role in determining whether GCT is ultimately delivered. In contrast, among patients residing in lower deprivation areas, age and functional status defined subsequent stratification in the primary model. Patients aged ≥80 years without spousal support had the highest rates of non-receipt, whereas, among younger patients, ECOG performance status further differentiated outcomes. In the sensitivity analysis excluding age, this structure was replaced by variables reflecting healthcare access and engagement and clinical status, including cancer screening, insurance, social support, and albumin, suggesting that multiple dimensions of clinical status contribute to treatment receipt in these patients.

These findings have direct clinical implications for early risk identification and targeted intervention. Patients residing in higher deprivation areas may benefit from system-level strategies focusing on improving care coordination at the point of entry, including expedited oncology referral, patient navigation, and coordination of multidisciplinary care at the time of initial presentation. In contrast, among patients in lower deprivation settings, non-receipt of GCT was more frequently associated with markers of clinical vulnerability, where early supportive interventions, including nutritional optimization, caregiver engagement, and proactive assessment of functional status, may help facilitate receipt of GCT.

Importantly, this analysis was restricted to variables available at the time of initial presentation to oncology care, reflecting an early point in the treatment pathway. As such, the observed patterns represent factors that may be identifiable at or near the first oncology visit, allowing clinicians to recognize patients at higher risk for non-receipt of GCT and consider early supportive or system-level interventions. These findings also suggest that distinct care pathways may be needed for patients with high versus low neighborhood-level deprivation, rather than a single uniform approach to improving receipt of GCT. Although a single model was used in this analysis, the observed divergence in stratification patterns between these groups suggests that future work may explore development of context-specific models tailored to different patient populations.

Patients with stage III–IV disease demonstrated higher rates of GCT than those with localized disease, reflecting differences in stage-specific treatment definitions rather than superior treatment delivery. Patients with localized PDAC generally require multimodality therapy, whereas systemic therapy alone may constitute GCT for locally advanced or metastatic disease. Disease stage was incorporated into outcome adjudication but intentionally excluded from CART model development, which was designed to identify potentially modifiable patient- and system-level factors present before definitive staging and multidisciplinary treatment planning.

The observed model performance (AUROC 0.662) is consistent with prior studies evaluating complex health services outcomes, which are influenced by both measurable clinical factors and unmeasured patient- and system-level factors.^18–20^ In this context, the purpose of this analysis was not to maximize predictive accuracy, but to describe clinically interpretable patterns of stratification for non-receipt of GCT. The identification of conditionally defined subgroups based on combinations of demographic, clinical, and socioeconomic factors may be informative for the development of future analytic approaches. By describing how these factors are conditionally related within specific patient groups, this approach may help guide selection of variables and interaction structures in subsequent analyses.^20^ The current analysis was restricted to variables available at the time of presentation to oncology care, reflecting an early point in the care pathway at which initial risk assessment may be possible. Future work incorporating longitudinal data may allow evaluation of how these relationships evolve over time and at later stages of care.

This study has several limitations. First, the retrospective design introduces the potential for residual confounding and misclassification. Although detailed review was performed, unmeasured factors such as patient preferences and provider decision-making may contribute to treatment selection and were not fully captured. Second, the outcome of interest, non-receipt of GCT, is inherently heterogeneous and may reflect a range of clinical, social, and system-level factors. Compared with receipt of GCT, non-receipt represents a smaller and more variable subgroup, which may limit model discrimination and complicate interpretation of predictive performance. Third, the study population represents patients who accessed care at tertiary referral centers. As such, this study may not capture individuals who were unable to access specialty care or who were lost earlier in the care pathway. Fourth, although classification trees identify clinically interpretable subgroups and hierarchical relationships, they are subject to instability, and splits may vary with sampling. Repeated cross-validation and sensitivity analyses were performed to address this, and the consistency of ADI as the initial stratifying variable across models supports the robustness of the observed structure. Finally, this study was conducted in the southeastern United States, and regional differences in healthcare delivery, access, and patient populations may limit generalizability to other settings. However, both institutions serve as referral centers and safety-net hospitals for a socioeconomically diverse population, providing representation of patients with a wide range of structural barriers.

Despite these limitations, this study has several strengths. The cohort includes a large and socioeconomically diverse patient population with substantial variation in access to care, enabling evaluation of both clinical and structural factors associated with treatment delivery. Detailed manual chart review of every patient's complete treatment course allowed rigorous adjudication of receipt of stage-appropriate therapy incorporating disease stage, anatomic resectability, multidisciplinary treatment recommendations, treatment sequencing, documented contraindications, disease progression, and patient goals of care. By focusing on variables available at the time of initial oncology presentation, this analysis reflects a clinically relevant timepoint in the care pathway for identifying patients at risk for non-receipt of GCT. The classification tree approach provides a complementary perspective to traditional regression models by describing conditionally defined subgroups in a clinically interpretable format. These findings may inform future work aimed at development of predictive models to identify patients at increased risk for non-receipt of GCT and evaluate targeted strategies to improve the delivery of GCT.

## Conclusion

In this multi-institutional cohort of patients with PDAC, non-receipt of GCT was associated with distinct patterns of stratification involving socioeconomic context, access to care, and clinical factors. Neighborhood-level deprivation consistently defined the initial partition of the cohort, with subsequent pathways reflecting differences in access to care and clinical vulnerability. These findings provide a clinically interpretable framework for factors associated with treatment delivery at an early timepoint in the care delivery pathway and will inform future efforts for the development of risk identification models and potential mitigating strategies to improve delivery of GCT.

## Supplementary Information

Below is the link to the electronic supplementary material.

Supplementary file1 (DOCX 18 KB)
