# Supplementary material for: Determinants of Non-Receipt of Guideline-Concordant Treatment in Pancreatic Cancer: A Classification Tree Analysis
Source: Ann Surg Oncol. 2026 Aug 12;33(12):10115–24. doi: 10.1245/s10434-026-20369-x (PMC13675330; doi:10.1245/s10434-026-20369-x)
Supplement: Supplementary file 1 [file 10434_2026_20369_MOESM1_ESM.docx]

**Supplementary Table 1. Primary reasons for non-receipt of guideline-concordant treatment (GCT) (N = 337)**

| **Primary Reason** | **No. of Patients** |
| --- | --- |
| **Health system and access barriers preventing receipt of GCT (N= 78, 23.1%)** | |
| Delays in diagnosis and/or treatment resulting in disease progression, clinical  deterioration, treatment attrition, or loss to follow-up | 25 |
| Lack of referral to Surgical Oncology despite resectable disease | 9 |
| Lack of referral to Medical Oncology | 10 |
| Social support limitations preventing treatment initiation | 12 |
| Insurance barriers | 5 |
| Clinical trial enrollment delay resulting in no systemic therapy and disease  progression | 1 |
| Incarceration related disruption of treatment | 3 |
| Substance use resulting in treatment interruption | 3 |
| Loss to follow-up after initial health system contact | 7 |
| **Treatment attrition/loss to follow-up after treatment initiation (N= 58, 17.2%)** | |
| Documented logistical, transportation, financial, or other barriers preceding  treatment discontinuation | 51 |
| **Failure to initiate recommended therapy (N= 46, 13.6%)** | |
| Recommended therapy not initiated; exact reason could not be definitively  determined after comprehensive chart review, however 38 patients with  documentation suggesting financial, logistical, or other barriers | 41 |
| Acute medical illness causing delays/preventing treatment initiation (e.g.,  perforated appendicitis, hip fracture, myocardial infarction, stroke) | 5 |
| **Clinical deterioration preventing initiation or completion of planned multimodality therapy (N= 147, 43.6%)** | |
| Deconditioning during systemic therapy preventing completion of planned  systemic or surgical therapy | 56 |
| Procedure-related complications resulting in deterioration and inability to  continue planned therapy | 39 |
| Upfront surgery without adjuvant chemotherapy due to postoperative  deterioration | 37 |
| Aborted upfront surgical exploration without subsequent systemic therapy | 15 |
| **Patient Preference (N= 8, 2.4%)** | |
| Deferred treatment ("not ready") without subsequent health system follow-up | 6 |
| Pursued alternative treatment instead of recommended therapy | 2 |
